# Advances in the study of HLA class Ib in maternal-fetal immune tolerance

**DOI:** 10.3389/fimmu.2022.976289

**Published:** 2022-08-29

**Authors:** Yiran Yang, Wanning Wang, Jing Weng, Huifang Li, Yanmin Ma, Lingyan Liu, Wei Ma

**Affiliations:** ^1^ Beijing Area Major Laboratory of Peptide and Small Molecular Drugs, Engineering Research Center of Endogenous Prophylactic of Ministry of Education of China, School of Pharmaceutical Sciences, Capital Medical University, Beijing, China; ^2^ School of Basic Medical Sciences, Capital Medical University, Beijing, China; ^3^ School of Traditional Chinese Medicine, Capital Medical University, Beijing, China; ^4^ Beijing Obstetrics and Gynecology Hospital, Capital Medical University, Beijing, China

**Keywords:** HLA class Ib, HLA-G, HLA-E, HLA-F, pregnancy, maternal-fetal immune tolerance, decidual immune cells

## Abstract

The HLA class Ib molecule is an alloantigen that causes transplant rejection on behalf of individual human and plays an important role in maternal-fetal immune tolerance. Early studies on HLA class Ib focused on the mechanism of HLA-G-induced immune escape, but in recent years, studies on the mechanism of HLA-G have deepened and gradually explored the mechanism of HLA-E and HLA-F, which are also HLA class Ib molecules. In the maternal-fetal interface, trophoblast cells express HLA class Ib molecules to protect the fetus from maternal immune cells by binding to inhibitory receptors of decidual immune cells (DICs) and shifting Th1/Th2 balance toward Th2 bias. Further studies on the molecular mechanism of HLA class Ib molecules provide a reference for its application in the field of clinical assisted reproduction.

## Introduction

Major histocompatibility complex (MHC) is a group of genes closely linked on the same chromosome, which is closely related to immune response and transplantation rejection. Human MHC is called Human Leukocyte Antigen (HLA) complex. HLA complex is located on the short arm of chromosome 6, with a total of 224 loci. It is divided into three regions according to the structure and function of each point gene and its coding product, namely class I, class II and class III gene region. HLA class I gene region contains non-classical HLA-E, F, G and other loci (1), the genes on which are called HLA class Ib genes (2). The probability of HLA being identical between two unrelated individuals is extremely small, and this variability leads to allograft rejection. As diploid organisms, humans have two different HLA inherited from both parents (3).

The embryo implantation process can be regarded as a semi-allogeneic transplant process. The embryo with paternal antigen will theoretically cause maternal transplantation rejection, which is contrary to the fact that it is not attacked by the maternal immune system before delivery (4). Therefore, it can be inferred that there is a special tolerance effect on the maternal-fetal interface to ensure the normal progress of pregnancy.

Maternal-fetal immune tolerance occurs at the maternal-fetal interface, which is composed of maternal decidua and chorion developed from the trophoblast of blastocyst. According to previous studies, it is generally believed that the immune tolerance of the maternal immune system to the fetus is related to the interaction between the immunotolerant microenvironment of the extravillous trophoblast and the deciduous layer. There are a large number of DICs in the immunotolerant microenvironment of the decidual layer to ensure the normal process of embryo implantation and spiral artery reconstruction in early pregnancy (5). DICs exist throughout pregnancy, and the population frequency varies with the stages of pregnancy. Decidual natural killer cells (dNK) account for the majority of DICs, followed by decidual macrophages and decidual T cells and so on. However, when maternal blood is in direct contact with syncytiotrophoblasts (STBs) in late pregnancy, DICs can enter the maternal-fetal interface and carry out immune rejection to the fetus (6–8).

In recent years, relevant studies have shown that HLA class Ib play an important role in maternal-fetal immune tolerance (9). In order to prevent the fetus from being attacked by the maternal immune system, extravillous trophoblasts (EVTs) express HLA class Ib molecules such as HLA-E, HLA-F and HLA-G to change the function of DICs and regulate its subtypes (7). Therefore, this review will elaborate on the comprehensive mechanisms and frontier applications in HLA class Ib, in the hope of offering new ideas for the diagnosis and treatment of pregnancy-related diseases.

## The HLA class Ib complex

The HLA class Ib genes include HLA-E, HLA-F and HLA-G, and are characterized as being non-polymorphic compared to their classical counterparts and encode molecules involved in immune regulation and immune suppression (2). The HLA class Ib genes expressed on the blastocyst, which developed from the fertilized egg and further differentiates into inner cell mass (ICM) and trophectoderm (TE) (7). TE further differentiates into epithelial trophoblasts (10). As one type of epithelial trophoblasts, cytotrophoblasts (CTBs) have two distinct differentiation pathways, generating syncytiotrophoblasts (STBs) and extravillous trophoblasts (EVTs) (11).

**Figure 1:**
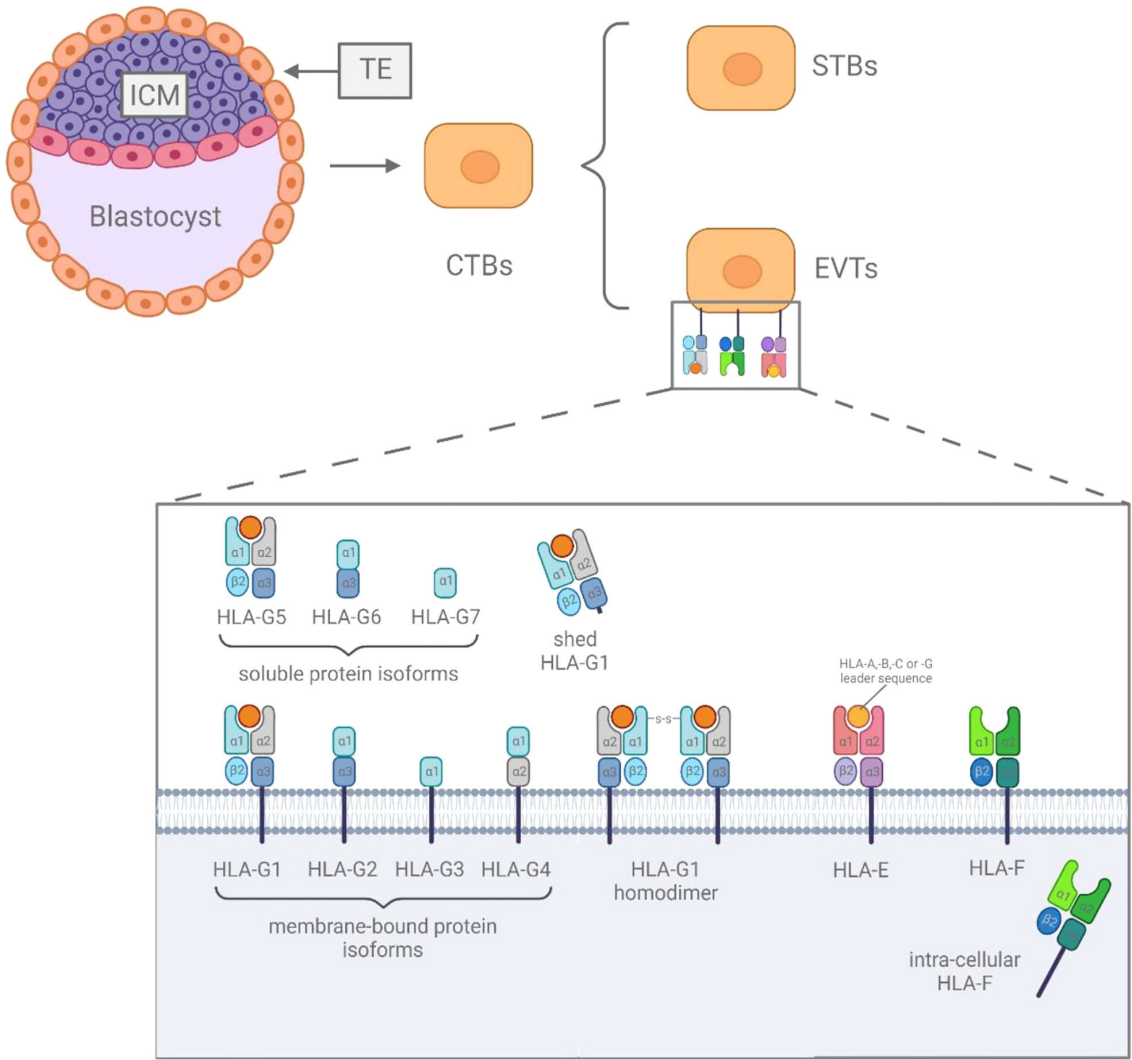
The difference among HLA class Ib.

The expression of HLA-E on the cell surface is regulated by the acquisition of peptides derived from the leader sequences of HLA-C and HLA-G molecules (12), and can be discovered in all stages of pregnancy. HLA-E was found to be expressed on the surface of EVTs (13), and it was weakly expressed in CTBs and STBs at 5 weeks of gestation (14). The expressions of HLA-F are less well understood, based on existing research results, it is still controversial whether HLA-F is expressed in cells or on the surface of cells (12). In fact, studies have shown that HLA-F was observed to be expressed on the surface of EVTs (14), and in the cytoplasm of CTBs, STBs and EVTs (15, 16). HLA-G is most abundantly expressed at immune-privileged sites (2), it can be found extensively expressed on the surface of EVTs during the entire pregnancy (12), and its soluble isoforms can be secreted by CTBs and STBs (**Figure 1**) (15, 17).

## Immunomodulatory effect of HLA-G

HLA-G is exclusively expressed in extravillous trophoblast cells (18, 19). It can be present in seven isoforms, four membrane-bound (m) isoforms (mHLA-G: HLA-G1, -G2, -G3 and -G4) and three soluble (s) isoforms (sHLA-G: HLA-G5, -G6 and -G7). In addition, the membrane-bound HLA-G1 can also be available in soluble form, named shed HLA-G1, which is mediated by metalloproteinase cleavage (20). Furthermore, there are HLA dimers linked by disulfide bonds, which have shown higher receptor affinity and slower dissociation rates in several studies (21, 22). In 1997, HLA-G was first proved to have a protective effect on the fetus after semi-allogeneic transplantation in the maternal immune system, which was also supported in pathological research by Yie et al (23). Nowadays, HLA-G is considered to be a major immune checkpoint molecule and plays a crucial role in maternal-fetal immune tolerance (24, 25). HLA-G can increase the activation threshold of immune cells before immune response by up-regulating the expression of inhibitory receptors on immune cells such as dNK cells and decidual T cells (26–28).

### HLA-G inhibits the killing effect of dNK cells

**Figure 2:**
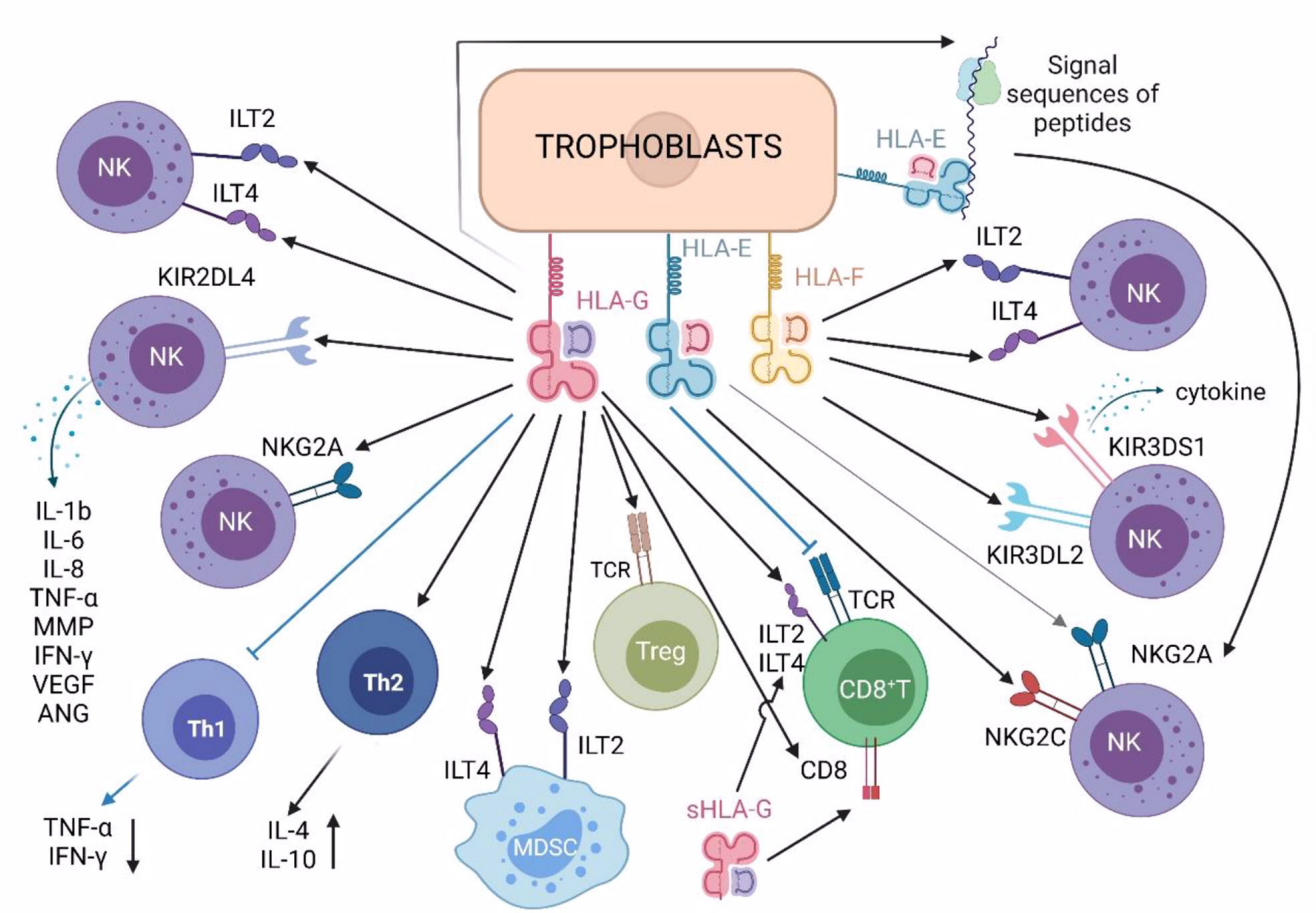
Roles of HLA class Ib molecules in immune cells in maternal decidua.

sHLA-G and mHLA-G recognize and bind killer cell immunoglobulin-like receptor KIR2DL4, immunoglobulin-like transcript 2 (ILT2), immunoglobulin-like transcript 4 (ILT4) and inhibitory receptor CD94/NKG2A of C-type lectin superfamily (**Figure 2**) on dNK cells, inactivating NK cell effectors (29–36). KIR2DL4 transduces inhibitory signals and is a killing inhibitory receptor on the surface of NK cells (37). HLA-G is the only known ligand of KIR2DL4 (38). The combination can transmit immunosuppressive signals, hence, to promote the development of a beneficial immune tolerance environment to the trophoblast/fetus, by protecting trophoblast cells from the killing of maternal dNK cells (39–44). In addition, the combination of HLA-G and KIR2DL4 can also promote dNK cells to secrete pro-inflammatory and pro-angiogenic factors (i.e., IL-1B, IL-6, IL-8, TNF-α, MMP, IFN- γ, VEGF, ANG). These cytokines ensure sufficient blood supply for the developing fetus and promote embryo implantation by invading the decidua and participating in the vascular remodeling of uterine spiral artery (**Table 1**) (45). ILT2 is expressed on the surface of T cells, B cells, monocytes/macrophages, dendritic cells (DC) and NK cells, can recognize the expression of HLA-G β-2 microglobulin. HLA-G inhibits the cytotoxicity of dNK cells by up-regulating the expression of ILT2 (26, 28, 46, 47). At the same time, the interaction between ILT2 and HLA-G can also inhibit the formation of NK-cell synapse and significantly reduce the ability of NK cells to kill target cells (**Table 1**) (28). ILT4 also binds HLA-G and plays a similar function to ILT2 (**Table 1**) (44, 45). CD94/NKG2A is another inhibitory receptor on the surface of dNK cells. It can recognize HLA-G1 (and HLA-E) expressed on trophoblasts, and negatively regulate the cytotoxicity of dNK cells (**Table 1**) (41, 48, 49).

**Table 1:** The recent studies of HLA class Ib in Reproductive immunology.

HLA class Ib	Related immune cells	Major receptor	Related cytokines	References
HLA-G	NK cell	KIR2DL4	IL-1B、IL-6、IL-8、TNF-α、MMP、IFN-γ、VEGF、ANG↑	(37–45)
		ILT2	——	(26, 28, 46, 47)
		ILT4	——	(44, 45)
		CD94/NKG2A	——	(41, 48, 49)
	CD8^+^ T cell	ILT2, ILT4	——	(50, 51)
		CD8	FasL	(50, 52)
	CD4^+^ Treg cell	——	——	(6, 40, 53, 54)
	——	——	IL-4↑, IL-10↑;TNF-α↓, IFN-γ↓	(55, 56)
	MDSCs	ILT2, ILT4	signal transducer activator of transcription 3 (STAT3)	(27, 44)
HLA-E	NK cell	CD94/NKG2A	——	(57–65)
HLA-F	NK cell	KIR3DL2	——	(66–68)
		LIR1,LIR2	——	(69–71)
		KIR3DS1	——	(72–74)

mHLA-G can even inhibit the cytotoxicity of NK cells by binding to the suppressive subset of NK cells (NK-ireg) and secreting inhibitory molecules (75).

### HLA-G exerts a wide range of immunosuppressive effects through trogocytosis

Trogocytosis (76) is a direct transfer of membrane and membrane-related molecules between cells in a contact manner (39). CD4^+^ T cells, CD8^+^ T cells, dNK cells and monocytes have been found that can obtain HLA-G through trogocytosis (45, 77). For example, CD4^+^ T cells can obtain HLA-G protein from decidual DC through trogocytosis and become the trogocytosis-based generation of temporary regulatory CD4^+^ HLA-G^acq+^ T cells (78–80). In addition, trogocytosis prolongs the effect of HLA-G on KIR2DL4-mediated signaling in dNK cells, increases the secretion of cytokines and other small proteins, and plays an important role in placental and fetal development, as well as the establishment of immune tolerance. Therefore, trogocytosis contributes to HLA-G-mediated extensive immunosuppression at the maternal-fetal interface (39).

### HLA-G inhibits the killing function of T cells

Studies have shown that both membrane-bound and soluble HLA-G proteins inhibited T cells alloproliferation (50). This inhibition involved engagement of ILT2 and ILT4 receptors by HLA-G (**Table 1**) (50, 51). Moreover, HLA-G can interaction with CD8 to enhance FasL expression, leading to apoptosis of CD8^+^ T cells (52), and can mediate cell-cycle inhibition of alloreactive T cells through Fas/FasL interaction (**Table 1**) (50), effectively inhibit the immune killing activity of cytotoxic T cells(CTL).

### HLA-G induces Treg cells to achieve immune tolerance

Treg cells protect the fetus after semi-allogeneic transplantation by binding with HLA-G (**Figure 2**), achieve maternal-fetal immune tolerance (53). Both mice and human cell experiments have shown that CD4^+^ CD25^high^ regulatory T cell population increases during pregnancy, and this trend can be observed as early as early pregnancy (54). At the same time, HLA-G has been found to have a direct induction effect on the enrichment of Treg cells (**Table 1**) (6, 40).

### HLA-G shifts Th1/Th2 balance toward Th2 bias

Normal pregnancy process tends to participate in humoral immunity with Th2-type cytokines and avoid cellular immunity with Th1-type cytokines. The imbalance of Th1/Th2 ratio can lead to maternal rejection of the fetus. HLA-G can promote the production of Th2-type cytokine interleukin-4 (IL-4) and up-regulate Th2-type cytokine interleukin-10 (IL-10) (55), and inhibit the synthesis of Th1-type cytokine interferon- γ(IFN- γ). HLA-G can also inhibit the tumor necrosis factor-α(TNF-α), which belongs to Th1-type cytokine. Thus, it can block the activation of NK cells, regulate placental growth and then maintain Th1/Th2 balance (**Table 1**) (56).

### HLA-G promotes the function of myeloid-derived suppressor cells (MDSCs) during pregnancy

MDSCs are innate immune cells, which increase during pregnancy. MDSCs simultaneously express ILT2 and ILT4 and inhibit the immune killing function of T cells (44). Natascha Köstlin et al. first described the direct effect of human sHLA-G on MDSCs (**Figure 2**). The study showed that sHLA-G induced MDSCs functionally and quantitatively through the signal transduction of ILT4 and the activation of transcription activator 3 (STAT3), reducing the positive rates of CD4^+^ T cells and CD8^+^ T cells (**Table 1**) (27).

## Immunomodulatory effect of HLA-E

In the process of reproductive immunity, HLA-E plays a protective role in the earliest events of implantation but not in active EVT invasion (19). HLA E mainly down-regulate the immune response at the maternal-fetal interface by cooperating with classical HLA class I molecules to ensure the success of pregnancy.

The expression of HLA-E is induced by many costimulators regulating classical HLA-I. One of HLA-E’s key functions is to regulate the activity of NK cells (81). In other words, the synergistic up-regulation of HLA-E and classical HLA-I is to protect target cells from NK mediated cytotoxicity. In an inflammatory environment in which many immune cells are recruited, this mechanism will protect bystander cells from immune system attacks while still allowing targeted destruction of some dysfunctional/virus infected cells (82). In the process of reproductive immunity, the immunological mechanism that determines the success of pregnancy mainly depends on the interaction between placental trophoblast and decidual immune microenvironment. HLA-E is an immunosuppressive factor, so the decrease of its expression level will weaken the down-regulation of immune response mechanism, leading to the termination of pregnancy (83).

HLA-E is of vital importance in the maternal immune process, because it can down regulate the maternal immune response, so as to protect the fetus from the attack of the maternal immune system. Among them, the interaction between HLA-E and NK cell CD94/NKG2A receptor is the most typical (**Table 1**) (57–59).

### Interaction between HLA-E and NK cell CD94/NKG2A receptor

NK cells can express a series of regulatory receptors related to their activation and inhibition, and selectively kill “non-self” components (84). HLA class I molecules are key regulators of NK cell activation. They regulate NK cell activity through interaction with inhibitory receptors and activated receptors. Therefore, HLA molecules are the key immune checkpoint of NK cells (85).

CD94/NKG2A heterodimer is an inhibitory receptor. It interacts with the trimer ligand which consists of HLA-E, β2m and a nonameric peptide. The receptor protein NKG2A can regulate the activity of NK cells, and CD94 plays a corresponding role after binding with the ligands (60).

HLA-E can interact with TCR on CD8^+^ T cells and regulatory T cells (61, 62), and with receptors NKG2A and NKG2C expressed on NK cells and some T cell subsets (**Figure 2**).Under physiological state, the engagement of CD94/NKG2A and HLA-E induced inhibitory signals that prevents NK cell activation (63). In other words, HLA-E can stimulate immune activation and inhibitory effects. When both NKG2A and NKG2C form heterodimers with CD94, NKG2A family members expressing inhibitory effects have more affinity for HLA-E than NKG2C receptors expressing activating effects (**Table 1**) (64, 65). For these two receptors, the results of receptor ligand interaction mainly depend on the level of HLA-E expression. In addition, P. Tripathi et al. investigated the HLA-E gene polymorphism of normal pregnant women and patients with recurrent spontaneous abortion. They found that the expression product of HLA-E^G^ allele had high affinity with the receptor and was highly associated with successful pregnancy (57).

In addition, HLA-E single chain trimer contains an additional (G4S)_3_ linker, which is fused with the peptide from HLA-G signal sequence into a non-polymorphic peptide, and presented by HLA-E. It can also inhibit NK cells dependent lysis by binding to CD94/NKG2A (81).

Norman Shreeve et al. obtained a relatively complete conclusion that maternal HLA-B/HLA-E/NKG2A pathway was conducive to healthy pregnancy and might have an impact on the health of offspring (59). By regulating this pathway, the occurrence of preeclampsia syndrome could be reduced, and abnormal pregnancy could be avoided.

In 2021, Eva Prašnikar and his team identified NKG2C/HLA-Eα2 domains and nonameric peptide were key elements involved in the molecular mechanism of signal transduction through intertwined hydrogen bond networks (86). Some studies also found that Ly49 gene family was involved in the synergistic regulation of the above process (87).

### HLA-E and HLA-G synergistically inhibit dNK cell activity

Because HLA-E can only be expressed in cells after binding to the signal peptide sequence derived from HLA-G (88), the expression of HLA-G was detected in all HLA-E expressing trophoblasts. Therefore, we speculate that HLA-G and E cooperate to inhibit the activity of dNK cells by binding inhibitory receptors, so as to protect trophoblasts from dNK cells killing, enable the maternal-fetal immune tolerance to embryonic alloantigens, and regulate the infiltration process of trophoblasts (88).

## Immunomodulatory effect of HLA-F

HLA-F was first discovered in 1990 (89), and there are few studies on the mechanism of HLA-F. The latest research showed that HLA-F could be expressed in an open conformation and bind to many Killer Cell Immunoglobulin Like Receptors (KIR receptors) on the surface of NK cells. They could also be expressed as HLA-F tetramers, binding to ILT2 and ILT4 (**Figure 2**) (69), which played a role in endometrial specific immune regulation during blastocyst implantation (90).

HLA-F protein is expressed by extravillous trophoblast cells with immunomodulatory properties and plays a role at the maternal-fetal interface (91). It was found that the expression of HLA-F protein could be detected on the cell surface of the extravillous trophoblast invading the maternal decidua (15, 92). HLA-F protein is related to the maternal-fetal immune tolerance to placental tissue. The EVT cell surface HLA-F in it interacts with the maternal immune cells of the decidua and protect the invading EVT from immune attack (14). HLA-F is expressed throughout pregnancy, and the expression increases with the passage of pregnancy time (93, 94). Its abnormally low expression (compared with healthy pregnant women) will lead to many diseases, such as Intrahepatic Cholestasis of Pregnancy (ICP), gestational hypertension, preeclampsia and gestational diabetes mellitus (19). These diseases are also closely related to low expression of HLA-G and HLA-E (95–99).

HLA-F is essential in the normal function of decidual trophoblast cells, as HLA-F is the third necessary partner of HLA-E and HLA-G in cell communication, while cell communication is the basis of pregnancy immunology. On this basis, studies showed that CD4^+^ CD25^+^ Treg mediated maternal tolerance to fetus (72, 100). These findings confirm that HLA-F is involved in the interaction between placental derived extravillous trophoblast and regulatory cells.

The variation of HLA-F and TAP2 genes leaded to shorter pregnancy time, indicating their role in endometrial specific immune regulation during implantation. The study also showed that HLA-F was necessary in maternal fetal immune regulation, which laid a foundation for the later study of the role of HLA-F in reproductive immunology. Moreover, any gene expression disorder in endometrium during pregnancy might lead to pregnancy failure. A variety of HLA molecules played an important and independent role at the maternal-fetal interface (101).

### Physical binding of MHC-I OC/HLA-F and KIR3DL2 inhibits the killing effect of uNK cells

Aura Burian et al. found that HLA-F and MHC-I open conformers(OCs) regulated the reactivity and specificity of KIR3DL2 to the target cells and effectors (66). KIR3DL2 receptor was an inhibitory receptor on the surface of NK cells (67). It can be activated after binding with HLA-F to produce immunosuppression, resulting in the weakening of Uterine nature killer (uNK) cells’ killing effect and the establishment of maternal immune tolerance to placental tissue (68). At the same time, because MHC-I OC and HLA-F are co-expressed, and the affinity between HLA-F and different MHC-I alleles is different, the expression level of HLA-F may be modified by allele MHC-I OC, and vice versa.

### The interaction of HLA-F tetramer with ILT2 and ILT4 receptors increases the activation threshold of immune effector cells

HLA-F has direct molecular interaction with ILT2 and ILT4 (69, 70). HLA-F might be a peptide binding molecule that can reach the cell surface and bind to the target peptide. On the cell surface, it can interact with LIR1 (ILT2) and LIR2 (ILT4) receptors to change the activation threshold of immune effector cells (69). During pregnancy, if CD4^+^ Th1 cells release Th1 cytokines, the immune response mainly mediated by cellular immune response will lead to pregnancy failure. Therefore, the expression of HLA-F in endometrial system, the expression of ILT2 and ILT4 on decidual T cells (**Table 1**) (71), and the interaction between HLA-F tetramer and ILT2 and ILT4 receptors, will reduce the immune rejection and protect the fetus from the damage caused by maternal immune rejection, so as to achieve the purpose of successful pregnancy.

### The opened conformations of HLA-F and MHC-I bind to NK cell Ig like receptor KIR3DS1 and activate the activity of uNK cells to some extent

The researchers experimentally confirmed the receptor ligand relationship between KIR3DS1 and MHC-I OCs - the activation of KIR might be mainly in the inflammatory response of up-regulated HLA-F and MHC-I OCs (**Table 1**) (73, 74). Then, some researchers proposed that HLA-F interacts with KIR3DS1 to activate NK cells (72). uNK cells are the main immune cells in uterine decidua (102). The contact between uNK cells and extravillous trophoblast cells is the first step of maternal immune recognition of placental tissue. Despite the strong lethality of uNK cells, the activation of uNK cells during pregnancy may be a necessary condition for the secretion of cytokines and growth factors, which are essential for the blood supply of the placenta.

### An association exists between HLA-F gene locus variation and pregnancy time and pregnancy success rate

All three SNPs in the HLA-F locus regulate the expression level of HLA-F in the secretory endometrium of patients with recurrent abortion (RPL), especially the direction of the A allele of rs2523393 SNP is associated with a better chance of pregnancy. At the same time, the functional basis of specific HLA-F single nucleotide polymorphism genotypes and diploids may lead to the increase of HLA-F mRNA and protein levels in some secretory endometrium, which may have an increased impact on embryo implantation and pregnancy development (91).

### HLA-F may be modified or interact with specific HLA-E receptors

The expression of HLA-F protein suggests that immune cells are activated (91). Takanori Shobu et al. suggested that the expression of HLA-E was similar to that of HLA-F. Their expression increased from the second trimester of pregnancy to full term, which was consistent with the time of rapid fetal growth. Both HLA-E and HLA-F might work together to prepare an environment that supports fetal growth (103). However, this was only confirmed by finding that the expression trend of HLA-E and HLA-F was similar and consistent with the time of rapid fetal growth.

In conclusion, HLA-Ib can be regarded as an immunosuppressive factor. When the expression of HLA increases, the maternal immune effect on embryos will be weakened, so as to achieve the purpose of successful pregnancy. However, how HLA-Ib expression is regulated has not been completely elucidated, and the polymorphism of the HLA Class Ib genes and proteins are very low. Therefore, when HLA-Ib expressed by the fetal trophoblast cells, they are unlikely to be seen as foreign by the maternal immune system. Furthermore, it is certain that when the body has an inflammatory reaction, the level of HLA in maternal serum will be affected (19). This indicates that the expression of HLA Ib is similar to other classic HLA molecules and will be affected by the inflammatory reaction. HLA-E, F and G not only play an independent role in the establishment of maternal-fetal immune tolerance, but also the synergistic effect of the three plays an important role. Abnormal expression of anyone will lead to pregnancy failure. In future, new discoveries may rise from the interaction mechanism between HLA-F and other MHC molecules, so as to understand the mechanism of maternal-fetal immune tolerance more deeply and lay a theoretical foundation for solving more reproductive immune diseases.

## HLA class Ib and pathological reproduction

Maternal-fetal immune tolerance has clinical significance for pathological conditions of pregnancy, which include recurrent spontaneous abortion (RSA), Pre-eclampsia (PE), repeated implantation failure (RIF), etc.

Several studies have reported that the differences of expression levels of HLA-G and alternative splice patterns are based on the 14-bp ins/del HLA-G polymorphism in exon 8 (the 3′UTR) (104). And the 14-bp ins HLA-G genotype associated with the low expression of HLA-G, and risk of RSA (104). However, recent studies have shown that HLA-G 14 bp insertion allele shows no significant association with RSA (45, 105, 106), deserving further research in the future.

PE is a multisystemic pregnancy disorder, which associated with the level of HLA class Ib (104). Several studies have reported significant reduced expression of HLA-G and sHLA-G in PE comparison with placentas from uncomplicated pregnancies. However, some studies have shown that the expression of HLA-G in the placenta did not observe any significant differences in expression intensity between cases of pre-eclampsia and controls (103, 104). The conflicting experimental results may arise from differences in the size, and the origin of the studied cohort, and may also be interfered by concurrent multiple diseases. In conclusion, the results remain controversial, and still need more research to figure out.

RIF is determined when transferred embryos fail to implant after several *in vitro* fertilization (IVF) treatment attempts (107). RIF may be due to the couple’s similarity in HLA components (107). If such a similarity is found, high-dose IV immunoglobulin should be offered before embryo transfer (108).

## Application of HLA class Ib in assisted reproduction

Based on the mechanism study of HLA class Ib molecules–HLA-E, HLA-F and HLA-G related to maternal-fetal immune tolerance, it can provide a new idea for the detection methods of *in vitro* fertilization-embryo transfer. At present, the research on the significance of HLA class Ib molecules for clinical embryo transfer mainly focuses on HLA-G (109, 110).

The latest research of Izabela Nowak et al. has made a great inspiration for HLA-G in the field of assisted reproduction. Their study found that the concentration of 59.73 IU/ml of sHLA-G had the best sensitivity (58.82%) and specificity (66.10%) to distinguish the successful pregnancy from the patients without pregnancy or abortion. At the same time, they concluded that the average and/or median concentration of sHLA-G after embryo transfer increased or at least remained at the level before embryo transfer (111). It suggested that the prediction of embryo transfer results could be accelerated by detecting the content of sHLA-G in maternal plasma samples, though the standard values of sHLA-G concentrations in plasma need to be supported by more experimental data. Venkatappa Vani et al. measured sHLA-G levels in embryo-spent medium (E-SM) samples and noticed that there was a positive correlation between sHLA-G levels and blastocysts’ grade scores, 60% of top-quality embryos have high level (3.86 ± 0.26ng/mL) of sHLA-G (112). Moreover, they showed a highly significant (P <.0001) association of sHLA-G with pregnancy outcome with live births, and the level of sHLA-G in E-SMs is a significant (P <.001) predictor of pregnancy outcomes (112).

Progesterone is known to be an immunomodulatory steroid hormone secreted by corpus luteum and placenta, which can maintain the endometrium and embryo implantation (113). Izabela Nowak et al. proposed that progesterone can induce the expression of HLA-G through progesterone response elements (111). Since the level of HLA-G was positively correlated with progesterone supplementation, exogenous progesterone might increase the expression of HLA-G (111). The Cochrane systematic review and the Practice Committee of the American Society of reproductive medicine also confirmed the important role of progesterone supplementation in luteal support for patients undergoing assisted reproductive technology surgery (114–116). Izabela Nowak et al. also mentioned that HLA-G was positively correlated with the supplement of corticosteroid and negatively correlated with estradiol, suggesting that the active and appropriate supplement of progesterone and corticosteroids during pregnancy, as well as the control of estradiol intake were conducive to the induction of HLA-G expression and beneficial in the maintenance and success of pregnancy (111).

Christina Bailey-Hytholt et al. innovatively invented a non-invasive cell enrichment technology. On a 50 degree inclined acrylic plate surface, they enhanced the adhesion of trophoblasts to the surface and limited the adhesion of cervical cells. They used gravity to make the cells move along the inclined plane, collecting trophoblasts in heterogeneous cervical cell populations, and identifying JEG-3 trophoblasts by HLA-G antibody staining and fluorescence imaging. It was confirmed that the purity of trophoblast in clinical cervical samples can be increased by 396 ± 52% (117). The design of the enrichment device can be used by non-professionals with low cost and high speed and can be directly integrated into the automatic cell picker instrument on the surface of the slide. The application of this technology in the detection of HLA-G content can speed up the detection of the success of embryo implantation and has a great application prospect in clinical detection.

## Discussion and prospects

As immune tolerance molecules expressed on the surface of trophoblast cells, HLA class Ib molecules has made a great contribution in the process of pregnancy, protecting the fetus from immune cells and maintaining normal growth. At present, the research on HLA-E and HLA-G has made the mechanisms and physiological functions clearer, but the research on HLA-F is still very few. Studies in recent years have basically confirmed its crucial role in maternal-fetal immune tolerance. However, the research on HLA-F is far from enough compared with HLA-E and HLA-G, and future experimental studies can focus on the role of HLA-F in physiological and pathological states. At the same time, some studies have proposed the synergy effects among HLA class Ib molecules, which can also be a new direction of future research. In addition, a deeper understanding of the mechanism of HLA class Ib molecules, especially HLA-G, and their expression in the extravillous trophoblast at the maternal-fetal interface can be used as a non-invasive biological index for rapid detection of embryo implantation. It can provide clues for maintaining normal pregnancy and further help to improve the success rate of embryo transplantation.

## Author contributions

Both YY and WW contributed to the study design and manuscript preparation and revision; JW and LL supervised the manuscript and provided financial support; HL and YM helped on literature collection; WM contributed to the overall supervision. All authors read and approved the manuscript.

## Funding

This work was financially supported by National Natural Science Foundation of China (No. 81901554), and Beijing Natural Science Foundation (No. 7222002).

## Conflict of interest

The authors declare that the research was conducted in the absence of any commercial or financial relationships that could be construed as a potential conflict of interest.

## Publisher’s note

All claims expressed in this article are solely those of the authors and do not necessarily represent those of their affiliated organizations, or those of the publisher, the editors and the reviewers. Any product that may be evaluated in this article, or claim that may be made by its manufacturer, is not guaranteed or endorsed by the publisher.

## Abbreviations

DICs, decidual immune cells; MHC, Major histocompatibility complex; HLA, Human leukocyte antigen; NK, nature killer; dNK, Decidual natural killer; uNK, Uterine natural killer; ICM, inner cell mass; TE, trophectoderm; CTB, cytotrophoblasts; STBs, syncytiotrophoblasts; EVTs, extravillous trophoblasts; mHLA-G, membrane-bound isoforms; sHLA-G, soluble isoforms; ILT, immunoglobulin-like transcript; DC, dendritic cells; CTL, cytotoxic T cells; IL-4 (10), interleukin-4 (10); IFN- γ, interferon- γ; TNF-α, tumor necrosis factor-α; MDSCs, Myeloid-derived suppressor cells; TCR,T cell receptor; MHC-I OC, MHC-I open conformers; RSA, recurrent spontaneous abortion; PE, Pre-eclampsia; RIF, repeated implantation failure; IVF, *in vitro* fertilization.
